# Effect of Traditional Chinese Medicine on the Gut Microbiota in Heat-Stressed Laying Hens

**DOI:** 10.3389/fvets.2022.905382

**Published:** 2022-06-21

**Authors:** Chunxin Ye, Qian Qu, Lin Bai, Jiaxin Chen, Zhuoke Cai, Jiaqi Sun, Cui Liu, Dayou Shi

**Affiliations:** ^1^Guangdong Polytechnic of Science and Trade, Guangzhou, China; ^2^College of Veterinary Medicine, South China Agricultural University, Guangzhou, China; ^3^Wens Foodstuff Group Co., Ltd, Yunfu, China

**Keywords:** traditional Chinese medicine, gut microbiota, heat stress, laying hen, 16S rRNA

## Abstract

Gut microbiota plays an important role in health and disease. To determine whether the traditional Chinese formula Zi Huang Huo Xiang San (ZHHXS) modulates gut microbiota under heat stress, a heat stress model was prepared in Roman layer hens by housing them at temperatures of 32–36°C and administering ZHHXS for 4 weeks. The Roman egg layers were randomly divided into three groups with 10 hens in each: a ZHHXS treatment group (ZHHXS-HS), a heat-stressed group (HS), and a blank control group (BC). The ZHHXS-HS and HS groups were housed in a 34 ± 2°C environment, while the BC group was housed at 25 ± 1°C. The ZHHXS-HS hens were fed a diet supplemented with 1% ZHHXS from 1 to 28 days, while the other groups were not. Gut microbiota in the hens' feces was assessed through 16S rRNA high-throughput sequencing on days 1, 3, 7, 14, and 28. A plot of the PCA scores showed that the gut microbiota composition in the BC group was a similar trend in the ZHHXS-HS group on days 1 and 3. The principal coordinate analysis (PCoA) unweighted distribution showed that the gut microbiota composition had no significant differences between the BC and ZHHXS-HS groups on days 1 and 7. The PCoA weighted distribution showed that the gut microbiota composition had no significant differences between the BC and ZHHXS-HS groups on days 1 and 3. This study showed that the composition of gut microbiota in layer hens with heat stress was modulated by ZHHXS treatment. ZHHXS treatment caused key phylotypes of gut microbiota to match the BC group, particularly *Actinobacteria, Bacteroidetes, Bacteroides*, and *Enterococcus*. The effect of ZHHXS in alleviating heat stress could be achieved by altering the composition of gut microbiota and regulating some key phylotypes.

## Introduction

Stress is a disorder associated with environmental factors, including temperature, diet, radiation, and so on. Heat stress is the most serious stress that causes adverse effects on growth performance, immunity, mortality, and breast meat quality (1–4). Emerging evidence demonstrates that heat stress can aggravate metabolic disorders and intestinal diseases (5–10). Based on our previous study, we found that heat stress has a critical impact on the composition of gut microbiota, growth performance, circulating levels of HSP70, and cortisol (11). Therefore, the gut microbiota may be the key factor in the treatment of heat stress.

The research has demonstrated that gut microbiota has responded and reacted to the treatment mechanisms of Traditional Chinese Medicine (TCM) (12–15). Gut microbiota is an important, complex, and substantial bacterial microecological system. Microorganisms in the intestine form a mutually compatible and beneficial harmonious relationship under long-term interactions with the organism. Under the mutual effect of the external environment, the host's health, and the bacterial microecological system, the balance with gut microbiota and the promotion of an organism's healthy development (16–18) can be maintained well. Gut microbiota, an ecological environment with a dynamic balance of organism and TCM, can regulate the overall balance of the body. Therefore, the regulation of TCM on the health of gut microbiota may be beneficial to balancing the body (19, 20). After Jia Wei Er Shu San was administered to weanling piglets, the diversity and structure of their gut microbiota significantly increased. The gut damage in a rat model with spleen deficiency was relieved through the regulation of gut microbiota *via* the decoction of four mild drugs. Gut microbiota in patients with type II diabetes can be regulated using a decoction of Ge Gen Huang Lian to treat diabetes (21). The turbulence of gut microbiota metabolites, such as short-chain fatty acids and hosts, was extensively modulated by Atractylodes macrocephala to achieve the therapeutic effects (22–24). Scutellaria baicalensis Georgi polysaccharide can improve intestinal barrier function and modulate gut microbiota on dextran sulfate sodium-induced ulcerative colitis (25). However, the study is required to further investigate the complexity of the interactions among TCM, heat stress, and gut microbiota.

In this study, the traditional Chinese formula of ZHHXS changed the diversity and structure of gut microbiota in layer hens under heat stress, and the change of some key phylotypes in gut microbiota may be responsible for the anti-heat stress effects of TCM.

## Materials and Methods

### Animals

Thirty 35-week-old Roman egg-laying hens were purchased from Yuan Shi Laying Hens Breeding Co., Ltd. (Guangzhou, China) and housed in standard environmental conditions. The ethical approval of the Animal Experiment Administration Committee of South China Agricultural University was obtained before the experiments began and all efforts were made to minimize the hens' suffering during the experiments. All the procedures involving the hens including their selection, management, and preparation throughout the experiments were conducted in strict accordance with Chinese legislation on the use and care of laboratory animals. The animals' housing, care, and handling were conducted at the Laboratory Animal Center of South China Agricultural University, Guangzhou, China.

### Preparation of Zi Huang Huo Xiang San

The traditional Chinese medicine formula used in this study was Zi Huang Huo Xiang San (ZHHXS) (Table 1). It was composed of eight dried Chinese herbs, namely Echinacea root, Scutellaria, patchouli, Elsholtzia, Gypsum, dried tangerine peel, white atractylodes rhizome, and licorice, which were mixed in the dry weight ratio of 4:4:3:3:2:2:1:1. The herbs were purchased from qualified suppliers based on standards specified in the *Chinese Pharmacopoeia* (Guangzhou, China). The herbs were crushed with a pulverizer and sifted using 80 mesh sieves. The materials were mixed to feed the hens for a basal diet supplemented with 1% of the mixture.

**Table 1:** The composition of the Zi Huang Huo Xiang San.

***Items***	***Chinese names***	**Actions**
*Echinacea root*	*Zi Zhui Ju Gen*	Strengthens immune system
*Scutellaria*	*Huang Qin*	Clearing heat, drying dampness, purging fire, and detoxification
*Patchouli*	*Guang Huo* *Xiang*	eliminating dampness with aromatics, stopping vomiting and relieving summer-heat
*Elsholtzia*	*Xiang Ru*	Diaphoresis, detumescence and removing dampness for regulating stomach
*Gypsum*	*Shi Gao*	clearing away heat and purging fire, except vexed, slake the thirst
*Dried tangerine peel*	*Chen Pi*	Regulating Qi and strengthening the spleen, drying dampness, and eliminating phlegm
*White atractylodes rhizome*	*Bai Zhu*	Strengthen the Spleen, dry up Dampness, tonify Qi, and prom
*Licorice*	*Gan Cao*	Strengthen the Spleen and tonify Qi, Harmonize the effects of other herbs

### Reagents

A TIANamp Stool DNA Kit (DP328) manufactured by Tiangen Biotech Co., Ltd. (Beijing, China) was used in this study. D1K ScreenTape and D1K Reagent manufactured by Agilent Technologies were also utilized. A Qubit dsDNA HS Assay Kit was purchased from Life Technologies. A TruSeq Custom Amplicon Sample Prep Kit and a MiSeq Reagent Kit v3 (600 Cycles PE) were purchased from Illumina.

### Experimental Design

Thirty Roman egg layers were randomly divided into 3 groups with 10 hens in each group: a heat-stressed group (HS), for which the heater temperature is maintained at 34 ± 2°C for 28 days, a ZHHXS treatment group (ZHHXS-HS), maintained at a temperature 34 ± 2°C with a basal diet supplemented with 1% ZHHXS from 1 to 28 days, and a blank control group (BC) maintained at a temperature 25 ± 1°C for 28 days. Hens in the HS and BC groups were provided a basal diet and free access to drinking water. The basal diet was shown in Table 2. The 200-mg feces were collected on days 1, 3, 7, 14, and 28. All feces samples were stored in a −80°C freezer until analysis.

**Table 2:** Composition and nutrient levels of the basal diet.

**Items**	**Content (%)**	**Nutritional level**	**Content (%)**
Corn	59.50	Crude protein	17.19
Wheat bran	4.00	Calcium	3.5
Soybean meal	14.60	Phosphorus	0.42
Cottonseed meal	6.00	Lysine	0.71
Rapeseed meal	4.00	Methionine	0.36
Dried meat floss	2.25	Cystine	0.19
Stone powder	8.4	Metabolizable energy (MJ/Kg)	12.82

### Bioinformatics Statistical Analysis

The sequence length, OTU (Operational Taxonomic Unit) numbers, and rarefaction curve (Chao1, Shannon, Good's coverage, and rank abundance) were performed using mothur on a single-summary command. Beta diversity analysis consisted of principal component analysis (PCA) and PCoA based on the Unifrac distance metric. The statistical analyses of the relative abundance of the phylum and genus levels, a taxonomy-based analysis, were carried out by analysis of variance (ANOVA) with SPSS 19.0. Values of *P* < 0.05 were considered statistically significant. The bar graph of the phylum and genus was produced with GraphPad Prism 5 software.

## Results

### The Sequence of Gut Microbiota

**Figure 1:**
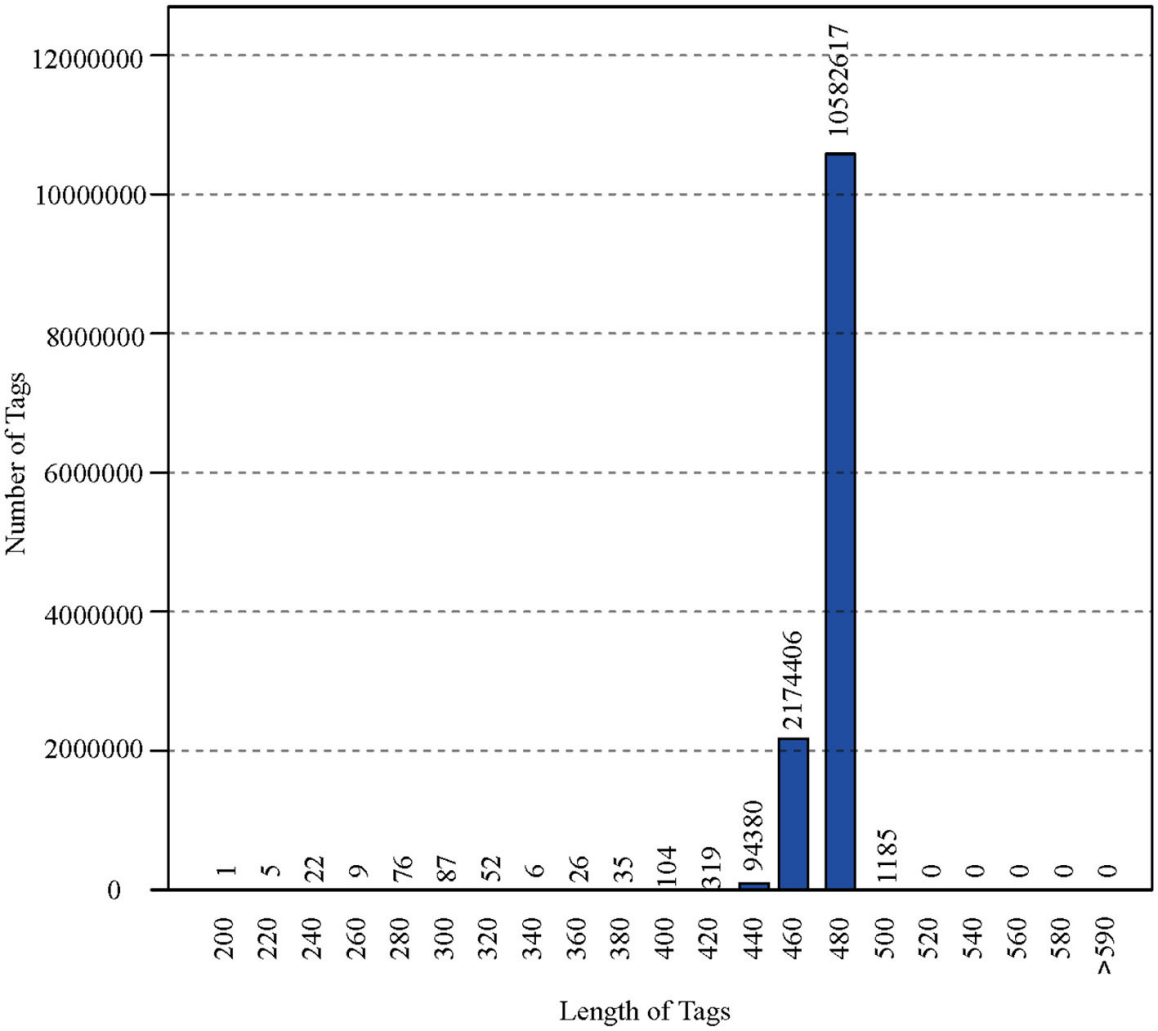
The length of the sequence and <200 bp were removed.

**Figure 2:**
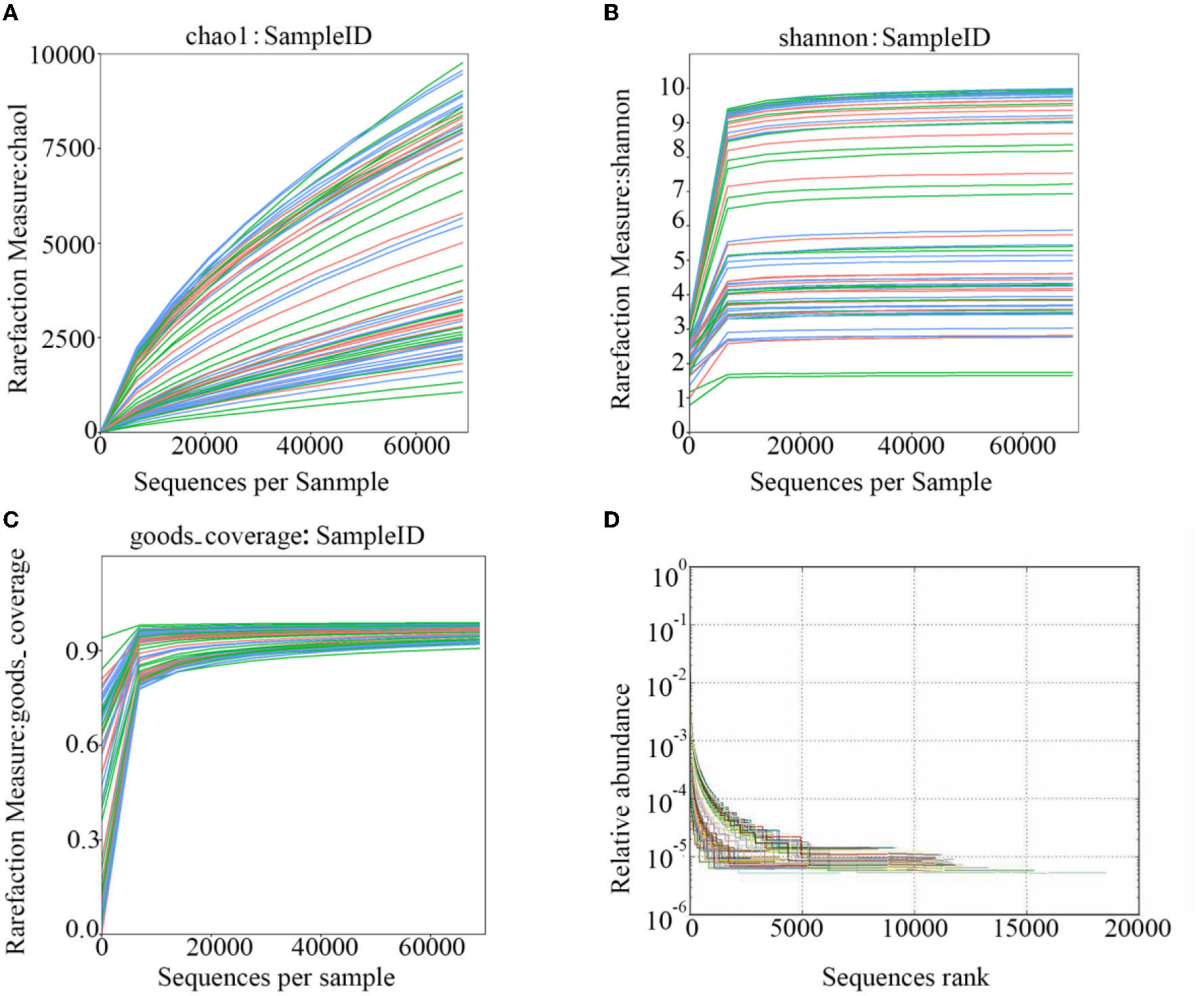
The rarefaction curves that were generated from the OTUs. **(A)** shows the chao1; **(B)** shows the Shannon; **(C)** shows the goods-coverage; and **(D)** shows the Rank-Abundance.

After polymerase chain reaction (PCR), all of the feces samples were sequenced by an Illumina MiSeq sequencer, which was used to monitor the structural changes in the three groups' gut microbiota. Sequence lengths of <200 bp were removed, 12,853,330 sequences were gained, and the average length was 480 bp (Figure 1). A total of 737,136 OTUs were generated through clustering analysis for high-quality sequences at a 97% similarity cut-off. The Chao1, Shannon, Good's coverage, and rank abundance curves that were generated from the OTUs suggested that high-sample coverage was captured with the sequencing depth (Figure 2), and further increases in the sequencing depth were unlikely to achieve greater gut microbiota diversity.

### The Gut Microbiota Composition Overtime After ZHHXS

**Figure 3:**
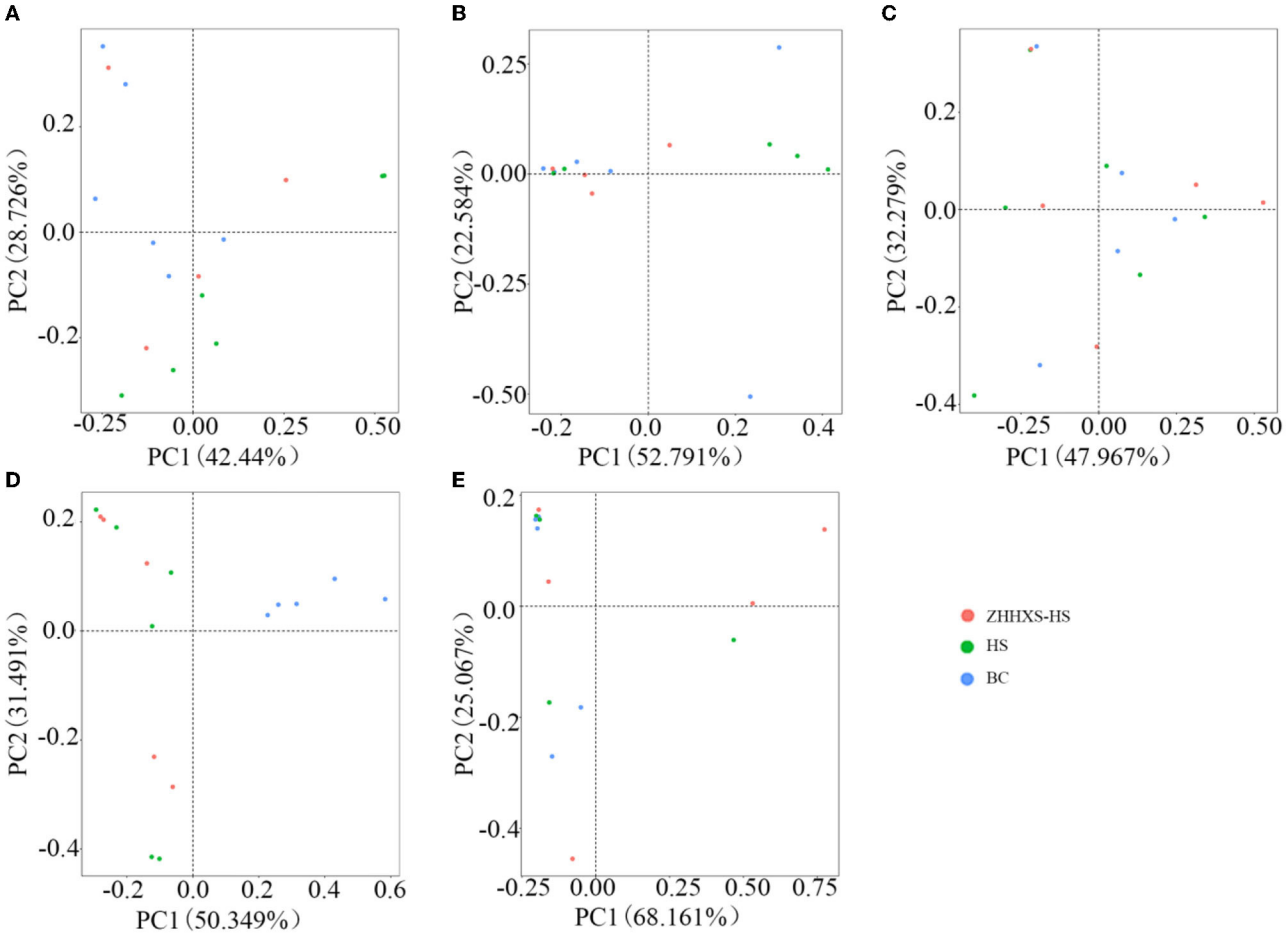
The structural changes of gut microbiota based on Principal component analysis (PCA). **(A)** The PCA on day 1 in different groups; **(B)** PCA on day 3 in different groups; **(C)** PCA on day 7 in different groups; **(D)** PCA on day 14 in different groups; **(E)** PCA on day 28 in different groups.

Principal component analysis, an unsupervised multivariate statistical method, was used to analyze the composition changes in the hens' gut microbiota (Figure 3). A plot of the PCA scores showed that the gut microbiota composition in the BC group was significantly different from the HS group along PC2 on day 1 and PC1 on days 3 and 14, and there was a similar trend in the ZHHXS-HS group on days 1 and 3. However, there were no significant differences among the BC, HS, and ZHHXS-HS groups on days 7 and 28.

**Figure 4:**
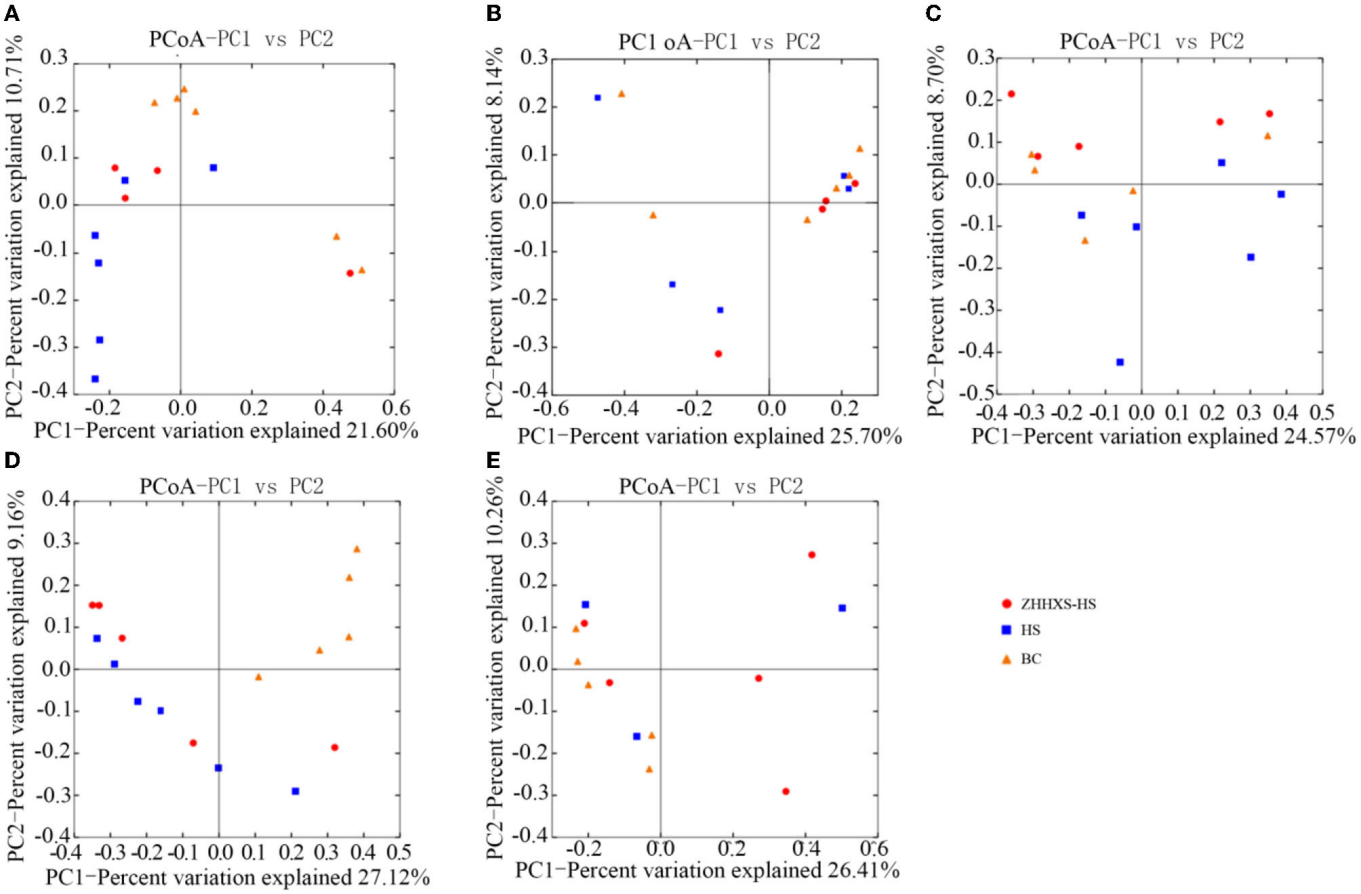
Changes of intestinal microflora composition based on unweighted principal coordinate analysis. The PCoA distribution with unweighting shows on day 1 **(A)**, on day 3 **(B)**, on day 7 **(C)**, on day 14 **(D)**, and on day 28 **(E)** in different groups.

**Figure 5:**
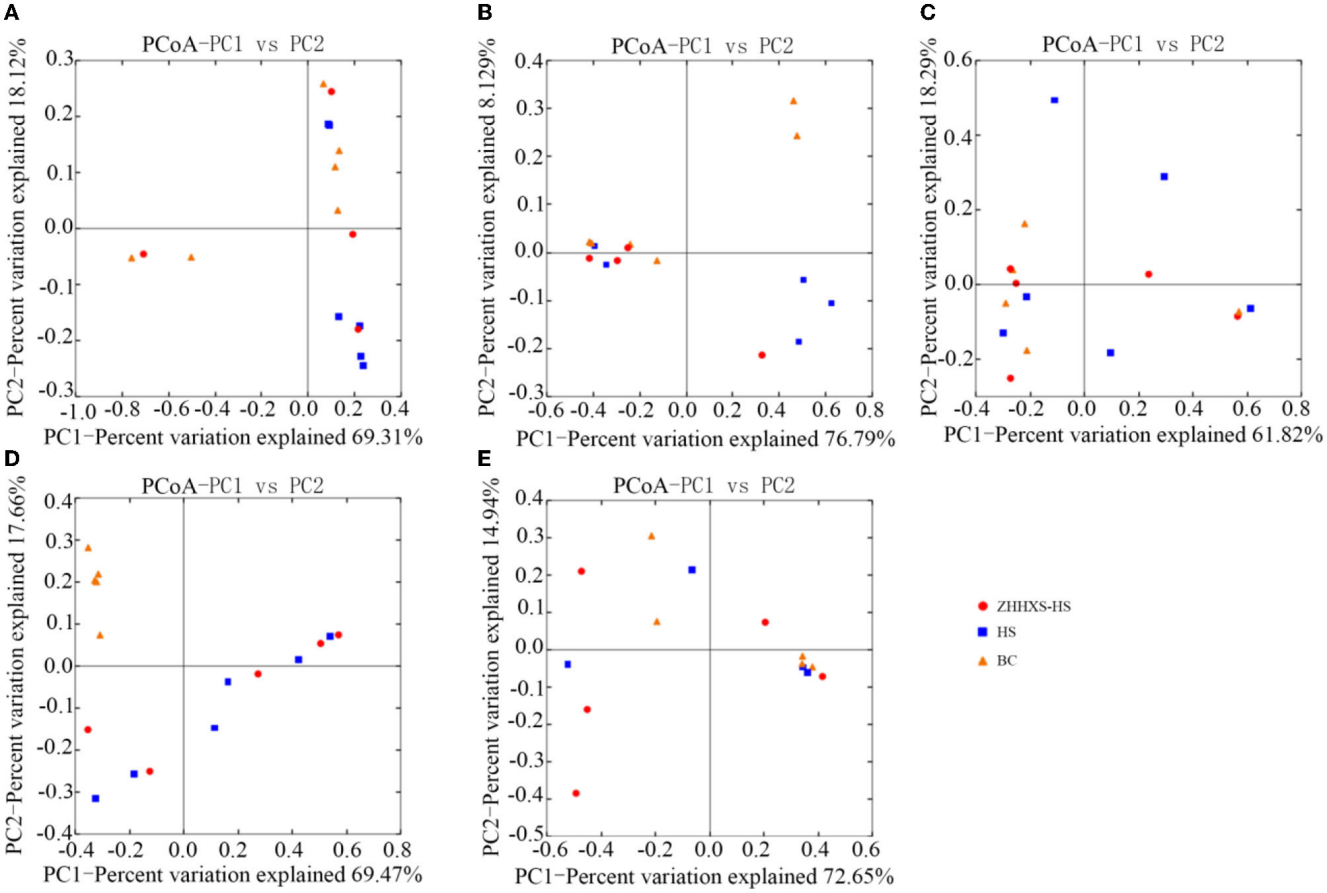
The composition changes of gut microbiota based on Principal coordinate analysis (PCoA) with weight. The PCoA distribution with weight shows on day 1 **(A)**, on day 3 **(B)**, on day 7 **(C)**, on day 14 **(D)**, and on day 28 **(E)** in different groups.

UniFrac distance-based PCoA, an unsupervised multivariate statistical method that includes weighted and unweighted distributions, was also used to analyze the gut microbiota composition (Figures 4, 5). The PCoA unweighted distribution showed that the gut microbiota composition in the BC group differed from the HS group along PC2 on days 1 and 7, and along PC1 and PC2 on days 14. However, there were no significant differences between the BC and ZHHXS-HS groups on days 1 and 7 and among the BC, HS, and ZHHXS-HS groups on days 3 and 28. The plot of the weighted PCoA showed comparable changes in the composition of gut microbiota between the BC and HS groups along PC2 on days 1 and 14, PC1 and PC2 on day 3, and no significant difference between the BC and ZHHXS-HS groups on days 1 and 3. There were no differences among the three groups on days 7 and 28.

### Key Phylotypes of Hens' Gut Microbiota Changed Due to ZHHXS

**Figure 6:**
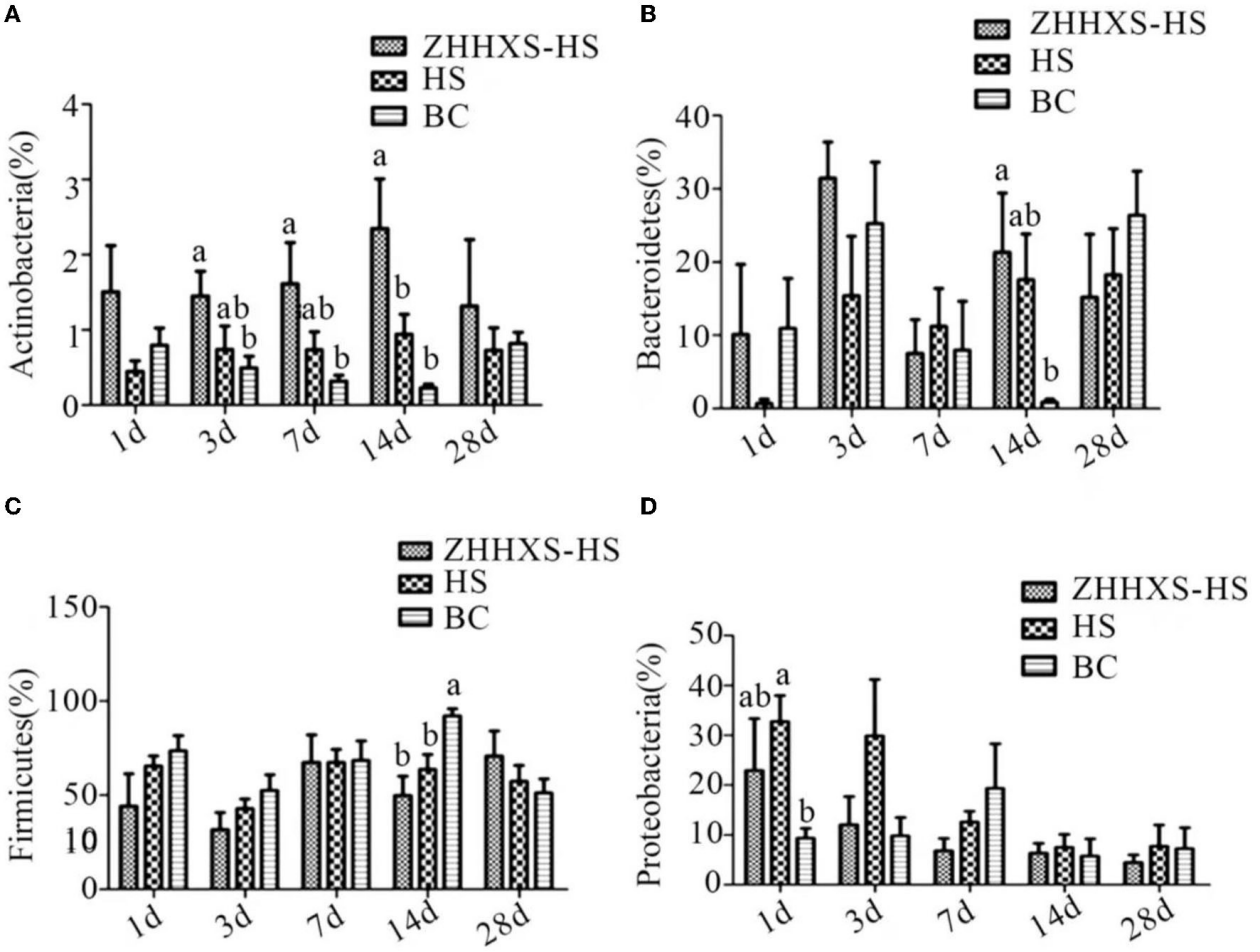
The changes of abundance at the phylum level through taxon-based analysis. **(A–D)** shows the abundance of *Actinobacteria, Bacteroidetes, Firmicutes*, and *Proteobacteria* among different groups across time, respectively. The significance of variance was analyzed by one-way ANOVA. The different letter represents *p* < 0.05 and there was an obvious difference.

Obvious changes in the key phylotypes of gut microbiota at both the phylum and genus levels were found through a taxon-based analysis among the three groups. At the phylum level, Firmicutes, Proteobacteria, and Bacteroidetes were the main groups (Figure 6). The relative abundance of Actinobacteria in the ZHHXS-HS group was markedly increased compared to the BC group on days 3, 7, and 14 and Bacteroidetes on days 14. There was no distinct difference between the HS and BC groups. The relative abundance of Firmicutes in the HS group differed significantly from the BC group on day 14 and Proteobacteria on day 1. The relative abundance of Firmicutes in the ZHHXS-HS group differed significantly from the BC group.

**Figure 7:**
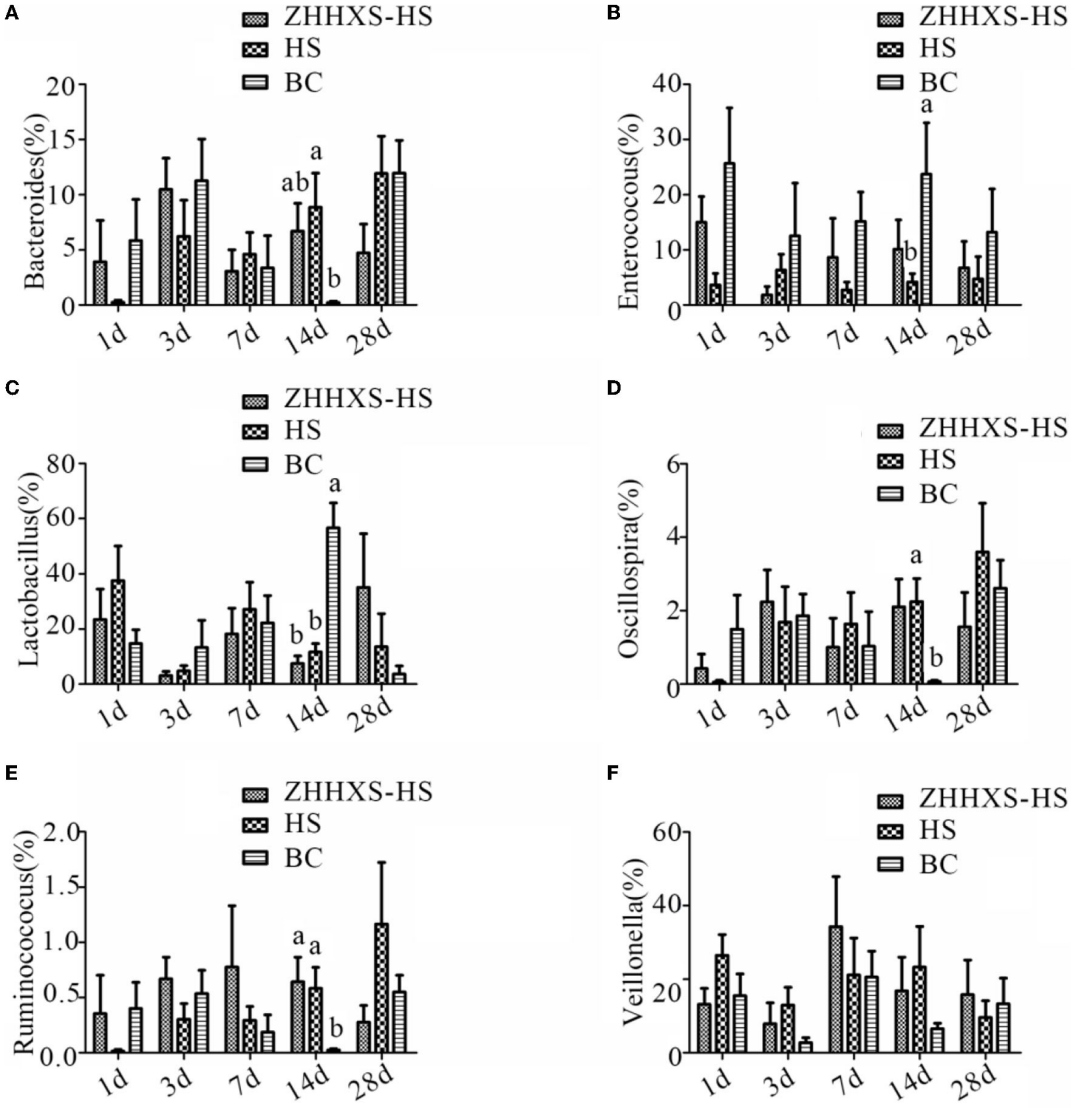
The changes of abundance at the genus level through taxon-based analysis. **(A–F)** shows the abundance of *Bacteroides, Enterococcus, Lactobacillus, Oscillospira, Ruminococcus*, and *Veillonella* among different groups across time, respectively. The significance of variance was analyzed by one-way ANOVA. The different letter represents *p* < 0.05 and there was an obvious difference.

A taxon-based analysis at the genus level revealed that *Lactobacillus, Veillonella, Enterococcus*, and *Bacteroides* were the dominant genus (Figure 7). The relative abundance of *Bacteroides* and *Enterococcus* changed between the HS and BC groups, and the ZHHXS-HS group had no significant change compared to the HS and BC groups on day 14. The relative abundance of *Lactobacillus* in the BC group significantly increased and *Oscillospira* and *Ruminococcus* decreased compared to the HS and ZHHXS-HS groups at day 14. There were no obvious changes between the HS and ZHHXS-HS groups. There were no marked differences among the three groups on days 1, 3, 7, and 28 in the six genera.

## Discussion

This study showed that gut microbiota differed among the ZHHXS-HS, HS, and BC groups. The results indicated that a traditional Chinese herbal formula is credible for heat stress. In agreement with this paper, other studies have reported that traditional Chinese medicine could treat heat stress (26–28). Scutellaria baicalensis, with the functions of clearing away heat and dampness, purging fire, and detoxification, has shown significant effects on the treatment of various diseases, especially hepatitis, diarrhea, vomiting, and high blood pressure (29).

The results of the study of the rarefaction curve showed that the sequencing depth was sufficient to cover the gut microbiota. The results also indicated that the composition of gut microbiota in the feces was markedly different among the ZHHXS-HS, HS, and BC groups. The PCA showed that the composition of gut microbiota in the BC group differed from the HS group and had a similar trend to the ZHHXS-HS group. The PCoA indicated that BC differed from the HS group, but there was no difference from the ZHHXS-HS group in the gut microbiota composition. However, the results of the PCA and PCoA differed at varying times. Other research has indicated that Chinese medicine compounds can change the composition of gut microbiota, although there is little similar research into heat stress (21, 30, 31).

Significant differences were observed at key phylum and genus levels. The relative abundance of Actinobacteria and Bacteroidetes was significantly increased in the ZHHXS group in this study. The results showed that the increased relative abundance of Proteobacteria caused by heat stress was reduced by ZHHXS. Firmicutes did not change with ZHHXS. This result is consistent with a report by Ying Chen that TCM can change the abundance of phylum (31). The relative abundance of *Bacteroides*, a short-chain fatty acid producer that is vital to the growth of gut microbiota (32, 33), increased due to heat stress compared to the BC group, and then decreased by ZHHXS. The changing abundance of *Enterococcus* was stabilized with ZHHXS. The growth promotion in fish and the multiple drug resistance of *Enterococcus* has been documented (34–36). These results further suggest that heat stress using ZHHXS may be mediated by changing the relative abundance of gut microbiota. However, there is no time-dependent change in gut microbiota by ZHHXS, which may be because gut microbiota is susceptible to another factor. Many other studies have shown that TCM can alter the abundance of the different genus to alleviate the severity of the disease (30, 31, 37). Atractylodes macrocephala Koidz. (called Baizhu in China) has long been used to treat gastrointestinal dysfunction. Crude extracts and pure compounds of Atractylodes macrocephala are used to treat gastrointestinal hypofunction and splenic asthenia (38). Pogostemonis Herba is usually used for the treatment of vomiting, abdominal pain, and diarrhea with the function of aromatic damp-resolving (39).

In the current study, the relative abundance of *Lactobacillus, Oscillospira*, and *Ruminococcus* was not correlated with ZHHXS and was positively correlated with a lack of treatment, suggesting that *Lactobacillus, Oscillospira*, and *Ruminococcus* might be phylotypes associated with the occurrence of heat stress. Heat stress with ZHHXS may be regulated by changing the relative abundance of *Veillonella*, although there was no significant variation among the three groups.

## Conclusion

This study suggested that the ZHHXS, a Chinese herbal formula, played a vital role in modulating gut microbiota during the treatment of layer hens with heat stress. By comparing the BC group, the ZHHXS-HS group showed that the relative abundance of Bacteroides and Enterococcus shifted to the same tendency. The relative abundance of Actinobacteria and Bacteroidetes became enriching through the ZHHXS on heat stress. So, the ZHHXS with the functions of clearing away heat and dampness, purging fire, and detoxification can modulate gut microbiota in heat stress. However, the causal relationship between Chinese herbal formula and gut microbiota remains unclear. This study provides evidence that the therapeutic effect of the Chinese herbal formula may function *via* the mediation of gut microbiota.

## Data Availability Statement

The original contributions presented in the study are included in the article/supplementary material, further inquiries can be directed to the corresponding author/s.

## Ethics Statement

All experimental procedures used in this study were approved by the Animal Ethics Committee of the South China Agricultural University (Guangzhou, China). The care and use of all animals were performed according to the Guidelines for Animal Experiments of the South China Agricultural University.

## Author Contributions

DS and CL conceived and designed the experiments. CY, QQ, and LB performed the experiments and collected and analyzed the data. CY and QQ wrote the article, while ZC, JS, and JC revised the article. All authors read and approved the final manuscript.

## Funding

This study was supported by the Natural Science Foundation of Guangdong Province (2021A1515011010) and the National Natural Science of Foundation (31672594 and 31602096).

## Conflict of Interest

LB was employed by Wens Foodstuff Group Co., Ltd. The remaining authors declare that the research was conducted in the absence of any commercial or financial relationships that could be construed as a potential conflict of interest.

## Publisher's Note

All claims expressed in this article are solely those of the authors and do not necessarily represent those of their affiliated organizations, or those of the publisher, the editors and the reviewers. Any product that may be evaluated in this article, or claim that may be made by its manufacturer, is not guaranteed or endorsed by the publisher.

## References

[1] St-Pierre NR Cobanov B Schnitkey G. Economic losses from heat stress by US livestock industries1. J Dairy Sci. (2003) 86:E52–77. 10.3168/jds.S0022-0302(03)74040-533398462

[2] Xu Y Lai X Li Z Zhang X Luo Q. Effect of chronic heat stress on some physiological and immunological parameters in different breed of broilers. Poult Sci. (2018) 97:4073–82. 10.3382/ps/pey256 29931080 PMC6162357

[3] Song ZH Cheng K Zheng XC Ahmad H Zhang LL Wang T. Effects of dietary supplementation with enzymatically treated *Artemisia annua* on growth performance, intestinal morphology, digestive enzyme activities, immunity, and antioxidant capacity of heat-stressed broilers. Poult Sci. (2018) 97:430–7. 10.3382/ps/pex312 29077887

[4] Cramer TA Kim HW Chao Y Wang W Cheng HW Kim Y. Effects of probiotic (*Bacillus subtilis*) supplementation on meat quality characteristics of breast muscle from broilers exposed to chronic heat stress. Poult Sci. (2018) 97:3358–68. 10.3382/ps/pey176 30137545

[5] Tsiouris V Georgopoulou I Batzios C Pappaioannou N Ducatelle R Fortomaris P. Heat stress as a predisposing factor for necrotic enteritis in broiler chicks. Avian Pathol. (2018) 47:616–24. 10.1080/03079457.2018.1524574 30221537

[6] Alhenaky A Abdelqader A Abuajamieh M Al-Fataftah AR. The effect of heat stress on intestinal integrity and Salmonella invasion in broiler birds. J Therm Biol. (2017) 70:9–14. 10.1016/j.jtherbio.2017.10.015 29108563

[7] Qu Q Li H Bai L Zhang S Sun J Lv W. Effects of heat stress on gut microbiome in rats. Indian J Microbiol. (2021) 61:338–47. 10.1007/s12088-021-00948-0 34290462 PMC8263838

[8] Yin C Xia B Tang S Cao A Liu L Zhong R. The effect of exogenous bile acids on antioxidant status and gut microbiota in heat-stressed broiler chickens. Front Nutr. (2021) 8:747136. 10.3389/fnut.2021.747136 34901107 PMC8652638

[9] Jadhav NV Awati B Kulkarni S Waghmare PG Suranagi MD Ravikanth K. Heat stress amelioration and production performance in layers supplemented with herbal liquid anti-stressor product. J Vet Med Anim Health. (2014) 6:69–74. 10.5897/JVMAH2013.0214

[10] He J He Y Pan D Cao J Sun Y Zeng X. Associations of gut microbiota with heat stress-induced changes of growth, fat deposition, intestinal morphology, and antioxidant capacity in ducks. Front Microbiol. (2019) 10:903. 10.3389/fmicb.2019.00903 31105682 PMC6498187

[11] Shi D Bai L Qu Q Zhou S Yang M Guo S. Impact of gut microbiota structure in heat-stressed broilers. Poult Sci. (2019) 98:2405–13. 10.3382/ps/pez026 30715508

[12] Wang HY Qi LW Wang CZ Li P. Bioactivity enhancement of herbal supplements by intestinal microbiota focusing on ginsenosides. Am J Chin Med. (2011) 39:1103–15. 10.1142/S0192415X11009433 22083984 PMC3349338

[13] Feng Q Liu W Baker SS Li H Chen C Liu Q. Multi-targeting therapeutic mechanisms of the Chinese herbal medicine QHD in the treatment of non-alcoholic fatty liver disease. Oncotarget. (2017) 8:27820–38. 10.18632/oncotarget.15482 28416740 PMC5438611

[14] Zhao L Nicholson JK Lu A Wang Z Tang H Holmes E. Targeting the human genome-microbiome axis for drug discovery: inspirations from global systems biology and traditional Chinese medicine. J Proteome Res. (2012) 11:3509–19. 10.1021/pr3001628 22624854

[15] Xu J Chen HB Li SL. Understanding the molecular mechanisms of the interplay between herbal medicines and gut microbiota. Med Res Rev. (2017) 37:1140–85. 10.1002/med.21431 28052344

[16] Cao ZJ Yu JC Kang WM Ma ZQ. [Research advances in the gut microbiota and inflammation in obesity]. Zhongguo Yi Xue Ke Xue Yuan Xue Bao. (2013) 35:462–5. 10.3881/j.issn.1000-503X.2013.04.020 23987498

[17] Guarner F Malagelada JR. Gut flora in health and disease. Lancet. (2003) 361:512–9. 10.1016/S0140-6736(03)12489-0 12583961

[18] Donaldson GP Lee SM Mazmanian SK. Gut biogeography of the bacterial microbiota. Nat Rev Microbiol. (2016) 14:20–32. 10.1038/nrmicro3552 26499895 PMC4837114

[19] Liu C Zhang C Lv W Chao L Li Z Shi D. Structural modulation of gut microbiota during alleviation of suckling piglets diarrhoea with herbal formula. Evid Based Complement Alternat Med. (2017) 2017:8358151. 10.1155/2017/8358151 29434646 PMC5757150

[20] Lv W Liu C Ye C Sun J Tan X Zhang C. Structural modulation of gut microbiota during alleviation of antibiotic-associated diarrhea with herbal formula. Int J Biol Macromol. (2017) 105:1622–9. 10.1016/j.ijbiomac.2017.02.060 28219687

[21] Xu J Lian F Zhao L Zhao Y Chen X Zhang X. Structural modulation of gut microbiota during alleviation of type 2 diabetes with a Chinese herbal formula. ISME J. (2015) 9:552–62. 10.1038/ismej.2014.177 25279787 PMC4331591

[22] Feng W Liu J Tan Y Ao H Wang J Peng C. Polysaccharides from Atractylodes macrocephala Koidz. Ameliorate ulcerative colitis via extensive modification of gut microbiota and host metabolism. Food Res Int. (2020) 138:109777. 10.1016/j.foodres.2020.109777 33288163

[23] Li C He L Dong H Jin J. Screening for the anti-inflammatory activity of fractions and compounds from Atractylodes macrocephala Koidz. J Ethnopharmacol. (2007) 114:212–17. 10.1016/j.jep.2007.08.002 17869038

[24] Amin A Hossen MJ Fu X Chou J Wu J Wang X. Inhibition of the Akt/NF-κB pathway is involved in the anti-gastritis effects of an ethanolic extract of the rhizome of Atractylodes macrocephala. J Ethnopharmacol. (2022) 293:115251. 10.1016/j.jep.2022.115251 35381310

[25] Cui L Guan X Ding W Luo Y Wang W Bu W. *Scutellaria baicalensis* Georgi polysaccharide ameliorates DSS-induced ulcerative colitis by improving intestinal barrier function and modulating gut microbiota. Int J Biol Macromol. (2021) 166:1035–45. 10.1016/j.ijbiomac.2020.10.259 33157130

[26] Dong H Zhong Y Liu F Yang K Yu J Xu J. Regulating effects and mechanisms of Chinese medicine decoction on growth and gut hormone expression in heat stressed pigs. Livest Sci. (2012) 143:77–84. 10.1016/j.livsci.2011.08.015

[27] Song X Xu J Wang T Liu F. Traditional Chinese medicine decoction enhances growth performance and intestinal glucose absorption in heat stressed pigs by up-regulating the expressions of SGLT1 and GLUT2 mRNA. Livest Sci. (2010) 128:75–81. 10.1016/j.livsci.2009.11.002

[28] Song X Luo J Fu D Zhao X Bunlue K Xu Z. Traditional Chinese medicine prescriptions enhance growth performance of heat stressed beef cattle by relieving heat stress responses and increasing apparent nutrient digestibility. Asian Australas J Anim Sci. (2014) 27:1513–20. 10.5713/ajas.2014.14058 25178304 PMC4150185

[29] Zhao T Tang H Xie L Zheng Y Ma Z Sun Q. *Scutellaria baicalensis* Georgi. (Lamiaceae): a review of its traditional uses, botany, phytochemistry, pharmacology and toxicology. J Pharm Pharmacol. (2019) 71:1353–69. 10.1111/jphp.13129 31236960

[30] Yin X Peng J Zhao L Yu Y Zhang X Liu P. Structural changes of gut microbiota in a rat non-alcoholic fatty liver disease model treated with a Chinese herbal formula. Syst Appl Microbiol. (2013) 36:188–96. 10.1016/j.syapm.2012.12.009 23453736

[31] Chen Y Xiao S Gong Z Zhu X Yang Q Li Y. Wuji Wan formula ameliorates diarrhea and disordered colonic motility in post-inflammation irritable bowel syndrome rats by modulating the gut microbiota. Front Microbiol. (2017) 8:2307. 10.3389/fmicb.2017.02307 29218037 PMC5703868

[32] Jumpertz R Le DS Turnbaugh PJ Trinidad C Bogardus C Gordon JI. Energy-balance studies reveal associations between gut microbes, caloric load, and nutrient absorption in humans. Am J Clin Nutr. (2011) 94:58–65. 10.3945/ajcn.110.010132 21543530 PMC3127503

[33] Mohd SM Sieo CC Chong CW Gan HM Ho YW. Deciphering chicken gut microbial dynamics based on high-throughput 16S rRNA metagenomics analyses. Gut Pathog. (2015) 7:4. 10.1186/s13099-015-0051-7 25806087 PMC4372169

[34] Leclercq R. Enterococci acquire new kinds of resistance. Clin Infect Dis. (1997) 24(Suppl. 1):S80–4. 10.1093/clinids/24.Supplement_1.S80 8994783

[35] Ghori I Tabassum M Ahmad T Zuberi A Imran M. *Geotrichum candidum* enhanced the *Enterococcus faecium* impact in improving physiology, and health of *Labeo rohita* (Hamilton, 1822) by modulating gut microbiome under mimic aquaculture conditions. Turk J Fish Aquat Sc. (2018) 18:1255–67. 10.4194/1303-2712-v18_11_02

[36] Landman D Quale JM. Management of infections due to resistant enterococci: a review of therapeutic options. J Antimicrob Chemother. (1997) 40:161–70. 10.1093/jac/40.2.161 9301980

[37] Yu J Guo J Tao W Liu P Shang E Zhu Z. Gancao-Gansui combination impacts gut microbiota diversity and related metabolic functions. J Ethnopharmacol. (2018) 214:71–82. 10.1016/j.jep.2017.11.031 29198875

[38] Zhu B Zhang QL Hua JW Cheng WL Qin LP. The traditional uses, phytochemistry, and pharmacology of Atractylodes macrocephala Koidz.: a review. J Ethnopharmacol. (2018) 226:143–67. 10.1016/j.jep.2018.08.023 30130541

[39] Xu F Cai W Ma T Zeng H Kuang X Chen W. Traditional uses, phytochemistry, pharmacology, quality control, industrial application, pharmacokinetics and network pharmacology of pogostemon cablin: a comprehensive review. Am J Chin Med. (2022) 50:691–721. 10.1142/S0192415X22500288 35282804

